# Inhibitors of protein translocation across membranes of the secretory pathway: novel antimicrobial and anticancer agents

**DOI:** 10.1007/s00018-017-2743-2

**Published:** 2018-01-05

**Authors:** Victor Van Puyenbroeck, Kurt Vermeire

**Affiliations:** Laboratory of Virology and Chemotherapy, Department of Microbiology and Immunology, Rega Institute for Medical Research, KU Leuven - University of Leuven, 3000 Leuven, Belgium

**Keywords:** Protein translocation, Signal peptide, Translocon, Endoplasmic reticulum, Translocation inhibitor, Sec61, SecY

## Abstract

Proteins routed to the secretory pathway start their journey by being transported across biological membranes, such as the endoplasmic reticulum. The essential nature of this protein translocation process has led to the evolution of several factors that specifically target the translocon and block translocation. In this review, various translocation pathways are discussed together with known inhibitors of translocation. Properties of signal peptide-specific systems are highlighted for the development of new therapeutic and antimicrobial applications, as compounds can target signal peptides from either host cells or pathogens and thereby selectively prevent translocation of those specific proteins. Broad inhibition of translocation is also an interesting target for the development of new anticancer drugs because cancer cells heavily depend on efficient protein translocation into the endoplasmic reticulum to support their fast growth.

## Different signal peptide-dependent translocation pathways

More than 30% of all human genes encode proteins destined for the extracellular environment, cell membrane or components of the secretory pathway [1]. Since protein synthesis occurs in the cytosol, translocation of proteins across biological membranes is essential for cellular function. Multiple pathways of protein translocation have been proposed. In general, translocation from the cytosol requires three key steps: (1) substrate recognition and targeting to the destination membrane, while maintaining the substrate in a translocation-competent state (2) translocation across or integration into that membrane, which usually requires energy expenditure in the form of GTP, ATP or proton motive force and (3) release, folding and maturation of the protein substrate.

Targeting signals contain the principal information that drives protein translocation. They direct newly synthesized proteins to their target membrane for translocation or membrane integration [2]. Signals present at the N-terminus of the synthesized protein are termed signal peptides (SP) or signal sequences. SPs are cleaved from the mature protein after translocation by the signal peptidase complex (SPC) and are characterized by a short (8–12 amino acids) hydrophobic segment, but their length and amino acid composition are highly divergent [3, 4]. Alternatively, targeting is facilitated by uncleaved amino-terminal signals termed signal anchors (SA). Signal anchors can act as a transmembrane segment and usually contain about 20 (or more) hydrophobic residues, a length required to physically span the approximately 3 nm wide hydrophobic interior of the phospholipid bilayer in an α-helical fold [5]. A third distinction is made for a class of proteins called tail-anchored membrane proteins (TA proteins), which have a single hydrophobic transmembrane region at their C-terminus that acts both as a targeting signal and membrane anchor [6].

The conserved Sec-dependent pathway is used for the translocation of most eukaryotic proteins [7]. The central component of this system is the heterotrimeric Sec61 translocon complex, also known as the SecY complex in bacteria and archaea [8]. It forms an aqueous channel in the endoplasmic reticulum (ER) membrane which allows protein transport across the membrane and it facilitates insertion of hydrophobic protein segments into the lipid bilayer. The Sec pathway operates in two major modes of translocation: (1) co-translational translocation couples the ribosomal protein synthesis directly to translocation through the channel, which efficiently uses the energy from mRNA translation in ribosomes to drive protein translocation across the membrane, (2) while post-translational translocation delivers completely synthesized polypeptide chains to the membrane which is best understood in fungi and bacteria. Mitochondrial, chloroplast and peroxisomal protein import, as well as specialized bacterial secretion systems (suggested reviews: [9–12]) are not discussed here, but the general concepts of signal peptide-dependent translocation (use of targeting signals and specialized protein-conducting channels) are conserved in these systems.

## Co-translational translocation

The process of co-translational translocation is a multistep sequence that depends on dynamic interactions between many factors. For most of these steps, natural and synthetic inhibitors have been discovered that usually affect translocation of a broad range of co-translational substrates. However, some compounds are able to operate in a signal peptide-selective way.

**Fig. 1:**
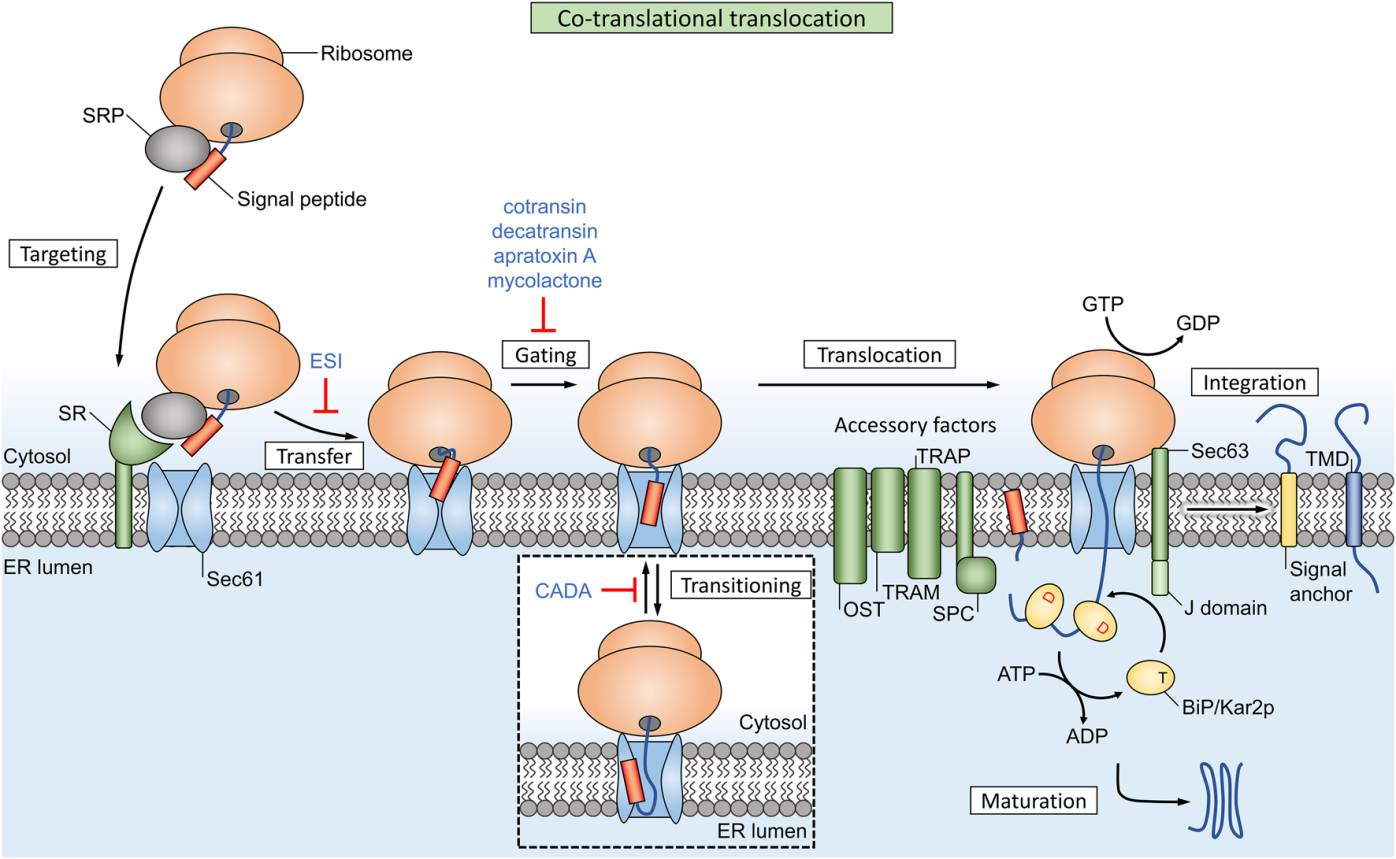
Co-translational translocation in eukaryotes relies on SRP for targeting to the Sec61 translocon. Ribosomal protein synthesis is coupled to translocation, which protects the peptide and effectively uses the energy from chain elongation as a driving force. The channel also facilitates membrane integration of hydrophobic transmembrane domains (TMDs, dark blue box) through a lateral gate. Multiple accessory factors reside in the local membrane environment and are dynamically recruited to assist the function of the translocon. The lumenal signal peptidase complex (SPC) cleaves signal peptides (orange box) from the mature protein, while uncleaved signal anchors (yellow box) function as transmembrane domains in integral membrane proteins. Inhibitors of co-translational translocation are indicated in blue text and the targets are explained in more detail in Table 1

Synthesis of proteins destined for the co-translational pathway starts with mRNA translation in cytosolic ribosomes (Fig. 1). Once the hydrophobic targeting signal of the nascent chain (NC) emerges from the ribosomal exit tunnel, it is recognized by the signal recognition particle (SRP), a universally conserved ribonucleoprotein complex [13]. The S-domain contains the evolutionarily conserved SRP54 subunit that binds signal peptides, functions as a GTPase and interacts with the membrane-associated SRP receptor (SR). Eukaryotic SRP has an additional Alu domain that forms an elongated, kinked structure. After recognition of the signal by the S-domain, the Alu domain reaches into the elongation factor binding site of the ribosome [13] and slows down elongation of the polypeptide chain. The SRP and ribosome-nascent chain complex (RNC) is then targeted to the ER membrane where SRP binds the SRP receptor (SR). The current model of SRP function suggests that this mechanism maintains the RNC in a translocation-competent state during the targeting step and GTP-dependent transfer of the RNC to the translocon [14, 15]. The Sec61/SecY translocon forms a passive pore in the ER membrane (or plasma membrane in bacteria) where the targeting signal needs to be recognized a second time. After successful recognition and opening of the pore, the polypeptide is finally translocated across or embedded into the membrane.

### The Sec translocon is a dynamic protein complex

The Sec61 complex consists of a central Sec61α subunit (referred to as SecY in bacteria and archaea) which forms the channel, and two smaller peripheral subunits Sec61β and Sec61γ [8]. Sec61γ is homologous to SecE in bacteria and archaea, but Sec61β shows little homology to the bacterial SecG subunit. Sec61α contains ten transmembrane helices divided into two halves of the channel (TM 1-5 and TM 6-10) with a hinge point between TM helix 5 and 6, often referred to as a ‘clam shell’ design. The ‘lateral gate’ of Sec61α, formed by the interface of transmembrane helices 2 and 7, allows opening of the channel towards the lipid bilayer for lipid insertion of transmembrane domains [16]. It also serves as the recognition site of signal peptides [17] and allows hydrophobic peptide region access to the lipid layer [18]. The inside of the channel is hourglass-shaped, with a ring at the center consisting of six bulky hydrophobic amino acid residues (the pore ring) which position their side chains to the center of the pore. This ring prevents leakage of ions through the inactive channel and during translocation of a protein substrate. The lumenal side of the closed channel is occupied by a short helix (TM2a) called the plug domain.

The translocon provides a dynamic interface between the water filled inside of the channel and the lipid environment. Hence, most eukaryotic membrane proteins with a (trans)membrane domain (such as a hydrophobic α-helix) are inserted into the ER membrane during co-translational translocation. In the current understanding of membrane integration, individual TM segments insert sequentially in the membrane layer through the lateral gate of Sec61 [19]. Furthermore, the channel can accommodate several TM helices at the same time, and facilitates early folding of these segments before release into the membrane [20]. Multiple accessory factors are dynamically recruited to the translocon and assist the Sec channel (Fig. 1), e.g., through the chaperoning functions of translocon-associated protein (TRAP) and translocating chain-associated membrane protein (TRAM). Other factors associate with the translocon to perform post-translational modification of the peptide substrates: oligosaccharyl–transferase (OST) complex facilitates N-linked glycosylation, while the SPC cleaves signal peptides from the mature protein.

### Non-selective inhibition of RNC transfer to the translocon

**Fig. 2:**
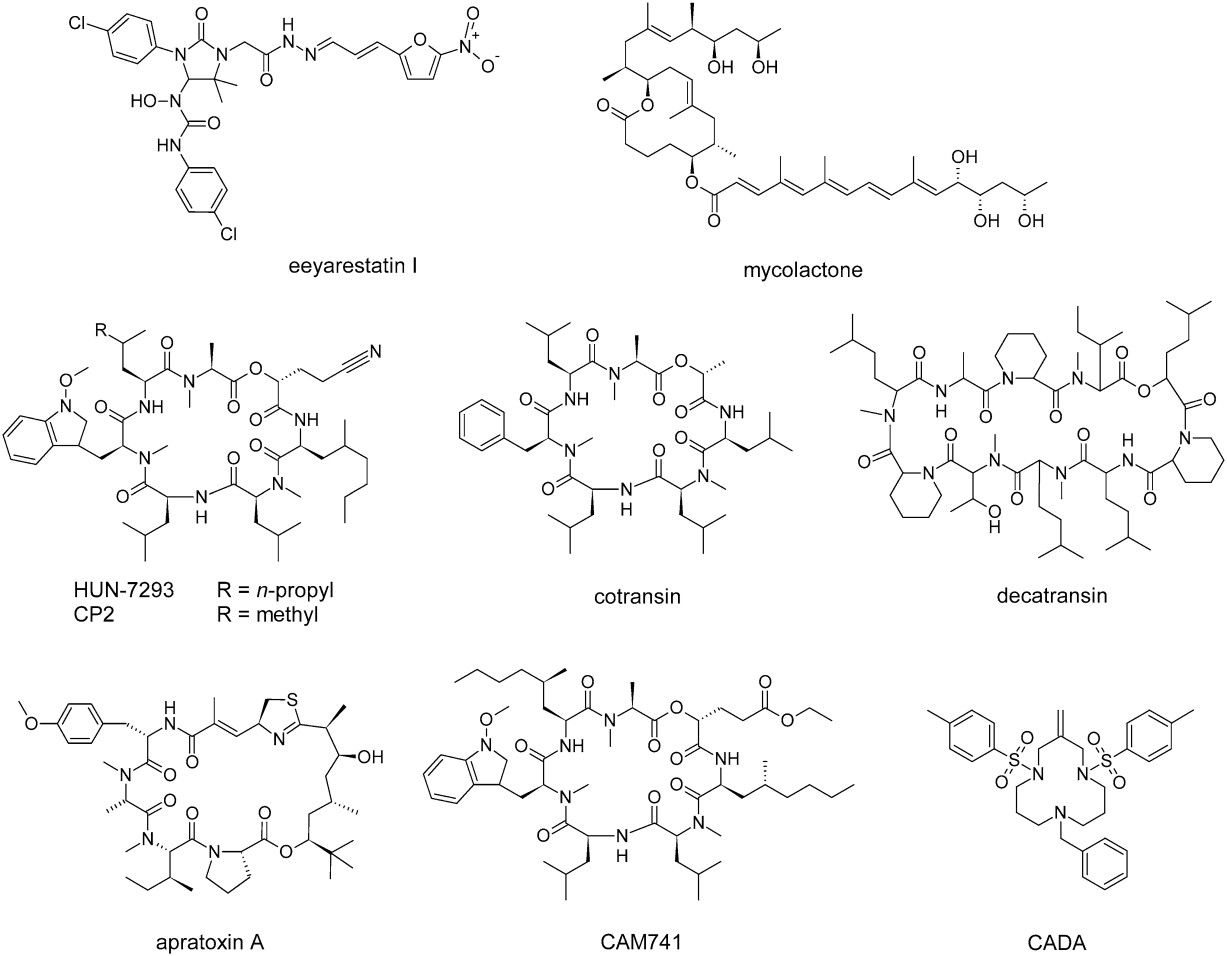
Chemical structure of several natural and synthetic inhibitors of co-translational translocation. Eeyarestatin I blocks the transfer of a SP from the RNC–SRP complex to Sec61. Mycolactone induces a conformational change in the Sec61 channel. HUN-7293 and its derivatives cotransin and CAM741 interfere with signal peptide insertion at the translocon. Decatransin and apratoxin A inhibit translocation into the ER lumen and can prevent growth of tumor cells. CADA prevents co-translational translocation of hCD4 and sortilin

The chemical compound eeyarestatin I (ESI) (Fig. 2) was discovered as an inhibitor of endoplasmic reticulum-associated protein degradation (ERAD) that induces an ER stress response in cells which leads to cytotoxicity [21]. ERAD removes misfolded proteins (and certain folded proteins) from the ER and plays a key role in ER homeostasis [22]. After recognition for ERAD, target proteins are modified by E3 ubiquitin ligases, retrotranslocated towards the cytosol and finally degraded by the proteasome. ESI inhibits the action of p97/VCP, a cytosolic ATPase that extracts polyubiquitinated ERAD substrates from the ER membrane [23]. ESI also interferes with co-translational protein import into the ER, as it blocks the transfer of a SP from the RNC-SRP complex into the Sec61 channel’s acceptor site [24]. This ESI-induced general block of protein translocation results in cytosolic accumulation of polyubiquitinated proteins and induces the unfolded protein response (UPR) [25]. The UPR normally protects the cell during ER stress, and is often upregulated in cancer cells. However, prolonged activation of UPR can induce cell death through apoptosis [26]. Consequently, ESI can be considered as an anticancer agent. In addition, Aletrari et al. showed that ESI also interferes with vesicular trafficking of Shiga-like toxin, which uses endosomal vesicles to reach the ER lumen and then exploits the ERAD machinery to enter the cytosol through ER retrotranslocation [27].

## Inhibitors of translocon gating

### Mycolactone

The polyketide macrolide mycolactone (Fig. 2) is a virulence factor produced by the human pathogen *Mycobacterium ulcerans* and causes necrotizing lesions of the skin without acute inflammation [28]. Hall et al. showed that mycolactone is a non-selective inhibitor of Sec61-dependent translocation across the ER [29]. Additionally, the inhibitory effect on cells was irreversible, indicative of a high affinity binding. The lack of immune response to this molecule is thus due to its suppression of inflammatory cytokine and receptor production in immune cells, and due to an indirect inhibition of antigen cross-presentation [30]. The eukaryotic Sec61 channel was recently identified as the target of mycolactone. Chemical crosslinking data suggest that the compound induces a conformational change of the channel that significantly disturbs co-translational translocation efficiency, but has less impact on post-translational translocation substrates [31].

### Exotoxin A

The *Pseudomonas aeruginosa* protein exotoxin A is a cytotoxic ADP-ribosyltransferase that enters the eukaryotic cytosol trough retrograde transport and inhibits retrograde export of immunogenic peptides from the ER towards the cytosol. It binds to Sec61α and prevents both co- and post-translational translocation [32, 33]. Exotoxin A also competes with cytosolic protein calmodulin (CaM) for binding to an N-terminal IQ motif on Sec61α and prevents Ca^2+^ leakage through the channel in human cells [34]. These observations suggest that the protein keeps the Sec61 channel in a closed state.

### Cotransins

A group of cyclic heptadepsipeptides are derived from the fungal macrocycle HUN-7293. The latter inhibits expression of three endothelial cell adhesion molecules: intercellular adhesion molecule 1 (ICAM-1), vascular cell adhesion molecule (VCAM-1) and E-selectin [35]. One derivative called cotransin (Fig. 2) was shown to inhibit the co-translational translocation of several proteins into the ER, in a signal peptide-selective way [36]. These initial studies reported inhibition of VCAM-1, P-selectin, angiotensinogen, β-lactamase, and corticotropin-releasing factor receptor 1 (CRF-R-1). Later studies also identified endothelin B receptor [37], human epidermal growth factor receptor 3 [38] and tumor necrosis factor alpha (TNFα) [39], a type II integral membrane protein with uncleaved signal anchor, as targets of cotransin.

Cotransin does not affect SRP recognition or targeting, but prevents access of NCs to the ER lumen, suggesting that the compound inhibits signal peptide-dependent gating of the Sec61 channel (Fig. 1). Accessory translocon factors such as TRAP, TRAM, Sec62/63 and binding immunoglobulin protein (BiP) are not required for cotransin activity, as the compound was able to selectively prevent translocation of VCAM-1 in minimal proteoliposomes (containing only Sec61 and SR) [36]. Garrison et al. suggested that cotransin either stabilizes the channel in a closed conformation or that it allosterically alters the signal peptide binding site of Sec61. These hypotheses, respectively, restrict productive interaction of low-affinity SPs or decrease the SP binding site flexibility, which both result in substrate selection at the translocon.

It must be noted that the reported compound concentrations used in the different translocation assays varies widely, which is important for the interpretation of the selectivity concept. For example, cotransin operates selectively at low nanomolar concentrations [36]. In contrast, Klein et al. have recently shown that a saturating concentration of cotransin (30 µM) actually inhibits translocation of a broad range of secreted proteins, while integral membrane proteins are mostly unaffected [40].

### Decatransin

Decatransin is a fungal cyclic decadepsipeptide (Fig. 2) that prevents growth of human carcinoma cells [41]. It is synthesized by a non-ribosomal peptide synthetase. Such very large modular enzymes are often used by microorganisms to produce complex secondary metabolites [42]. Decatransin prevents Sec61/SecY-dependent co- and post-translational translocation into the ER lumen but does not affect SRP recognition or SR targeting [41].

### Apratoxin A

Apratoxins are natural secondary metabolites isolated from a marine cyanobacterium. They are also produced by a non-ribosomal peptide synthetase [43]. The cyclic depsipeptide apratoxin A (Fig. 2) was discovered as a cytotoxic antitumor drug and prevents growth of various cancer cell lines by inducing G1 cell cycle arrest and apoptosis [43, 44]. Proteomic data showed that expression of a subset of secreted and membrane proteins are downregulated by apratoxin A, and this effect was due to an inhibition of their co-translational translocation [45].

## Gating inhibitors likely operate through a common mechanism

### Mutations in Sec61 provide cross-resistance to gating inhibitors

**Fig. 3:**
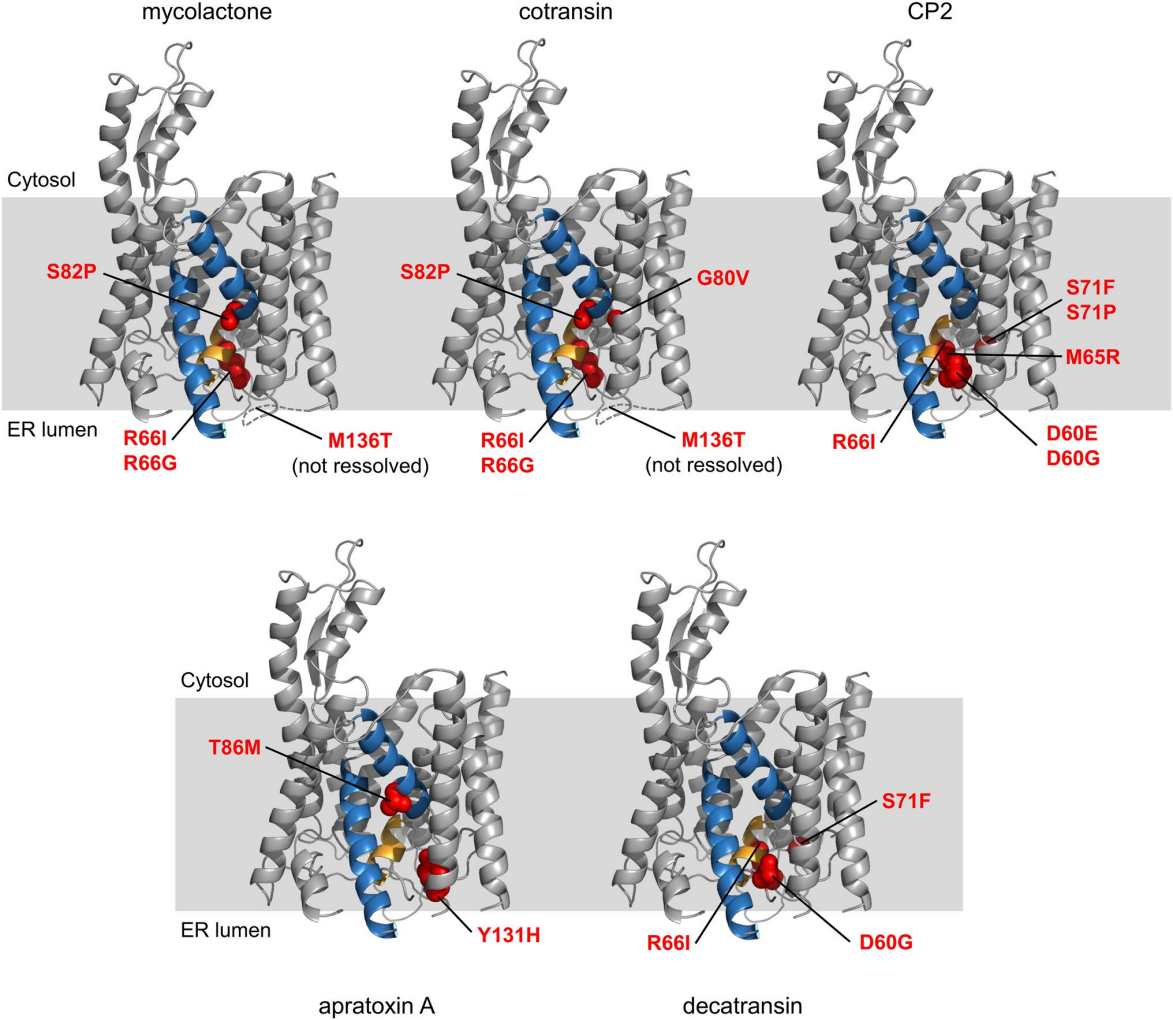
Location of the mutations in mammalian Sec61 that confer resistance to different gating inhibitors. Cryo-electron microscopy model of mammalian Sec61 from Protein Data Bank accession code 3J7Q [52]. The lateral gate (TM 2 and 7) is shown in blue and the plug helix in orange. The loop that connects TM 3 and 4 contains M136 but was not resolved in the model and is inferred from the Archaeal SecY structure (PDB 1RHZ) [8]

Photo-crosslinking experiments showed that HUN-7293 derivatives bind to the Sec61α subunit [46], as predicted earlier. A more recent study used genetic selection in the DNA repair-defective HCT-116 tumor cell line to identify mutations that confer cotransin resistance [47]. These mutations, located in a region near the lateral gate and plug domain of Sec61α, stabilize the plug domain in the closed state and allosterically prevent the translocation substrate from opening the channel (Fig. 3). Extensive crosslinking experiments showed that the arrested transmembrane domain (TMD) of TNFα is positioned at the interface of Sec61alpha helices 2 and 7, perpendicular to the channel helices. However, TMDs with increased hydrophobicity or helical propensity are able to escape the cotransin-induced block [47].

Accordingly, resistance mutations for apratoxin A were all located near the Sec61 plug domain and several of these mutations confer cross-resistance against cotransin [48]. Apratoxin A also competes with cotransin for translocon binding, suggesting a mutually exclusive binding site near the lumenal plug region. Compared to cotransin, apratoxin A does not arrest the TNFα TMD in a preferred orientation on the cytosolic side of Sec61. This suggests that the compound blocks TNFα translocation in an earlier step, before TMD insertion occurs [48].

Decatransin might act similar to cotransin, as several (but not all) mutations in Sec61 that provide resistance to cotransin also confer decatransin resistance. Additionally, both decatransin and CP2, a compound very similar to HUN-7293/cotransin, inhibit translocation in yeast Sec61α and bacterial SecY [41], suggesting that the translocon might contain a universal binding site for natural translocation inhibitors. Junne et al. hypothesized that these hydrophobic, peptide-like molecules mimic signal peptides and can bind to the Sec translocon, where they block incoming peptides and stabilize the closed translocon conformation [41].

Mycolactone showed a broad effect not only on co-translational translocation but also dose-dependently competed with a cotransin variant (CT7) for translocon binding suggesting that they likely share a binding site [49]. When tested for cross-resistance in the cotransin-resistant cell lines, several mutations near the lumenal plug indeed provided mycolactone resistance (Fig. 3).

### Gating inhibitors stabilize the translocon in a closed state

The *prl* (protein localization) phenotype of bacteria and yeast, characterized by the ability to translocate defective signal peptides, has historically been associated with mutations in the translocon plug and pore ring, as these mutations destabilize the closed translocon conformation [50, 51]. Interestingly, the observed resistance mutations for HUN-7293 derivatives, decatransin, apratoxin and mycolactone also map to residues located on the lumenal side of the translocon lateral gate, near the plug domain and thus resemble the *prl* phenotype. The resistance mutations are believed to increase the flexibility of the translocon, which overrules the action of the inhibitors, i.e., keeping the translocon in a closed state [41]. Paatero et al. suggested that these natural and synthetic compounds all target a similar binding site on the Sec61α translocon to regulate translocation (Fig. 3). Due to significant differences in their chemical structure and potency though, each of these natural compounds produces a distinct inhibitory profile [48].

Since the translocon machinery evolved to accept a broad range of targeting signals, it is surprising to see a selective inhibition of only a small subset of translocation substrates for some of the inhibitors. Nevertheless, such a selective suppression of cell surface receptor expression can have many novel therapeutic and antiviral applications. Hegde and Kang proposed that, in absence of accessory translocon components, the interaction between Sec61 and most signal peptides is intrinsically unstable [53]. Only a limited set of ‘strong’ sequences are able to engage the channel on their own, for a sufficiently long duration to allow for complete insertion of the elongating chain, and subsequent translocation of the protein. All other targeting signals (‘weak’ sequences) are assumed to have a very low basal translocation efficiency. In this model, the occurrence of many Sec61-associated protein complexes (e.g., TRAP, TRAM, Sec62/63, BiP and OST) that dynamically assist the translocon, is the key concept that regulates substrate-specific translocation efficiency. Selective inhibition of translocation is possible too, either through (allosteric) destabilization of channel/signal peptide interactions or through stabilization of the translocon channel in a closed state. Additional regulatory factors likely exist, as the current knowledge is mostly obtained from a very limited set of model translocation systems (e.g., dog pancreas cells, model yeast and bacterial systems). Properties of Sec-dependent signal peptides are thus linked to the observed variation in selectivity (the inhibitory profiles) of these gating inhibitors.

## Selective inhibition of signal peptide topology inversion

The small molecule cyclotriazadisulfonamide (CADA) is a synthetic macrocycle (Fig. 2) that showed antiviral activity against a broad range of human immunodeficiency virus (HIV) strains and human herpes virus-7 (HHV-7). Analysis of receptor expression showed that CADA treatment induced a downregulation of the cell surface- and intracellular CD4 levels. Since both HIV and HHV-7 use CD4 as the primary receptor for cell entry, this down-modulation of CD4 is responsible for CADA’s inhibition of viral entry [54].

CADA selectively inhibits the cell surface expression of human CD4 (hCD4) on a post-transcriptional level. The compound interacts with the signal peptide of hCD4 and it prevents co-translational translocation of the pre-protein chain across the ER membrane [55]. Instead, the affected precursor protein chains end up in the cytosol where they are degraded by the proteasome. Targeting of RNCs to the ER translocon is not affected by the compound. The hCD4 signal peptide initially inserts head-on (N_exo_/C_cyt_) into the translocon, and inverts to a looped topology (N_cyt_/C_exo_) upon chain elongation (transitioning, Fig. 1). Early models assumed that signal peptides generally insert into the translocon with a looped topology, but head-on insertion and a dynamic topology inversion has been described for signal anchor proteins [56, 57]. Vermeire et al. suggest that CADA interferes with the mandatory inversion (transitioning) of hCD4’s signal peptide, and thereby prevents translocation of the chain.

Furthermore, the down-modulating effect of CADA seems to be selective for hCD4 and the membrane glycoprotein sortilin. As previously shown for hCD4, CADA also inhibits the co-translational translocation of sortilin in a signal peptide-dependent way [58]. The effects of CADA on sortilin were less pronounced as compared to hCD4 though, and sortilin appears to be a secondary substrate of CADA. Importantly, expression of the homologous mouse CD4 protein is not affected by CADA. Mutagenesis of the CD4 signal peptide identified the central hydrophobic signal peptide region as crucial for CADA sensitivity, with a lesser contribution from the C-terminal SP region [55].

The binding site of CADA is not known, but quantitative structure–activity relationship studies of CADA analogues suggest a two-site binding model for these compounds [59, 60], and surface plasmon resonance experiments showed a selective, non-covalent interaction between CADA and the human CD4 signal peptide [55].

Another HUN-7293 derivative, CAM741 (Fig. 2), also interferes with the co-translational translocation of only a limited set of substrates in a signal peptide-selective way. VCAM-1 [61, 62] and VEGF [63] are reported CAM741 targets. Blocked polypeptide chains are directed towards the cytosol and degraded by the proteasome [36, 61]. Their cytosolic accumulation also induces the unfolded protein response [25]. The compound CAM741 prevents correct insertion of the VCAM-1 SP into the translocon [62]. Using a diagnostic amino-terminal glycosylation tag, the topology of the VCAM-1 SP was determined during the early post-targeting phase: it initially inserts head-on (N_exo_/C_cyt_) into the translocon channel and reorients upon polypeptide chain elongation. However, the amino-terminus does not enter the ER lumen in the presence of CAM741. Systematic analysis of VCAM-1 signal peptide mutants identified residues in the SP C-region, h-region and the first residue of the VCAM-1 mature domain as crucial elements for full CAM741 sensitivity [62]. VEGF-1 mutagenesis revealed a different pattern, as mutagenesis of leucines in the N-terminal SP region and hydrophobic residues in the h-region resulted in a loss of compound sensitivity [63].

Signal peptides with increased hydrophobicity are able to escape CAM741 activity, while reducing the hydrophobicity of VCAM-1 and VEGF-1 SP’s h-region increases the inhibitory effect of CAM741 and vice versa [62, 63]. Hydrophobicity is a major determinant of TM segment recognition at the translocon [64], and signal peptide recognition at the translocon is also dependent on hydrophobic interactions. Targeting signals were shown to interact with a specific hydrophobic patch on the cytosolic side of Sec61, after which they intercalate between the channel helices [18]. Mutations in the signal peptide that increase its hydrophobicity are thus better at opening the channel (they are “stronger” signal peptides according to Hegde’s theorem of translational regulation [53]) and this should allow them to overcome the translocational block imposed by CAM741 more easily.

## SRP-independent translocation

After more than 30 years of study, it is clear that SRP-dependent co-translational translocation is highly efficient. However, it is now also evident that a significant fraction of all ER-targeted proteins do not utilize the SRP pathway for targeting, and/or do not even rely on the Sec61 translocon for translocation [65–67]. Membrane proteins with large translocated domains or multiple TM domains are usually constrained to the SRP-dependent co-translational pathway, but other physical limitations can restrict SRP recognition. In bacteria and yeast, SRP recognition requires targeting signals with sufficiently high hydrophobicity. SPs with low or moderate hydrophobicity are targeted towards the Sec61-dependent post-translational pathway [68]. Mammalian SRP does not differentiate between SPs of different hydrophobicity, while microorganisms likely favor post-translational translocation to support higher rates of protein secretion, as this pathway does not use up the limited pool of ribosomes [69].

**Fig. 4:**
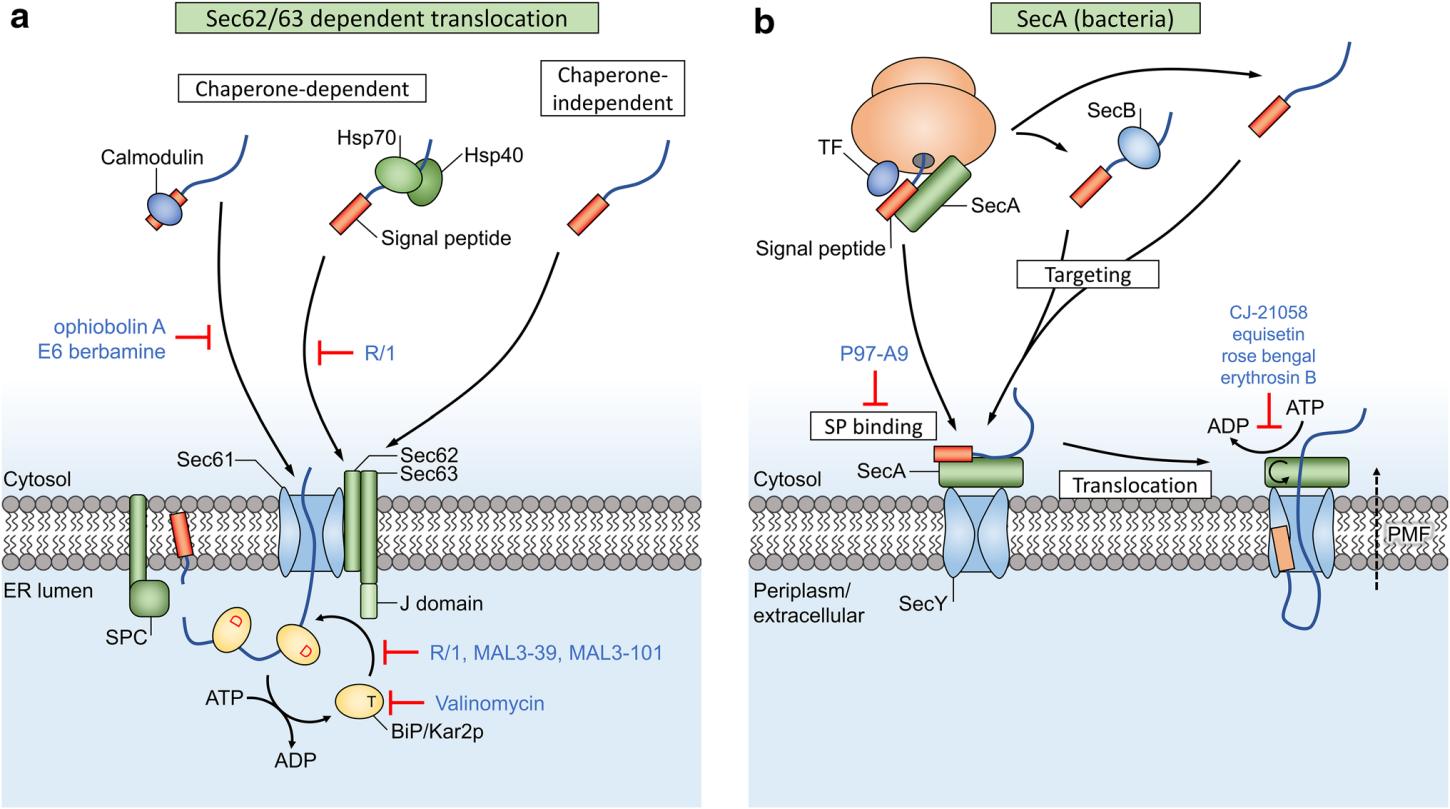
Sec-dependent post-translational translocation of pre-proteins involves several chaperone proteins. **a** Calmodulin binds signal peptides (orange box) and is targeted to Sec61, as Sec61 contains a cytosolic signal peptide-binding site. Hsp chaperones retain cytosolic proteins in a translocation-competent state and are targeted to Sec62/63. Other chaperone-independent targeting pathways are possible too, e.g., with intrinsically disordered proteins. The J domain of Sec63 converts BiP to a state with high affinity for protein binding. Sequential binding of BiP molecules works as a ratcheting mechanism that drives post-translational translocation. **b** Targeting of bacterial pre-proteins can occur either in a chaperone-dependent way through the help of SecA, SecB or trigger factor (TF), or in a chaperone-independent way. SecY-dependent post-translational translocation in bacteria relies on the essential ATPase motor protein SecA and the proton motive force (PMF) to drive translocation of pre-proteins through the SecY pore and across the plasma membrane. Inhibitors of translocation are indicated in blue text

These SRP-independent polypeptides must often be kept in a translocation-competent state during the targeting towards the translocon. The translocon pore is quite narrow and only allows passage of proteins with limited secondary structure. Therefore, excessive folding or aggregation of pre-proteins must be prevented (Fig. 4). Multiple cytosolic proteins, e.g., heat shock protein 70 (Hsp70) and Hsp40 chaperones, assist this process after completion of translation [70]. Hsp70 s bind to exposed hydrophobic protein regions in an ATP-dependent way, while their ATPase activity is regulated by Hsp40 co-chaperones. It is still unclear which factors target these chaperones towards the ER [66].

Another one of these cytosolic factors is the calcium-binding protein calmodulin, that was shown to maintain the small protein preprocecropin A (64 amino acids) in a translocation-competent state inside the cytosol, by selective binding to the signal peptide [71]. CaM also binds to the cytosolic N terminus of Sec61α, which is proposed as the targeting mechanism for these peptides.

## Chaperone inhibitors

### NSC 630668-R/1

In a screen for heat shock cognate 70 (Hsc70, a member of the Hsp70 chaperone family)-interacting compounds, NSC 630668-R/1 (referred to as R/1) was identified as an inhibitor of Hsc70 ATPase activity (Fig. 4). R/1 almost completely inhibits in vitro post-translational translocation of pre-pro-α-factor (ppαF) in yeast microsomes at a concentration of 300 µM, with an IC_50_ of 6 µM [72].

### Calmodulin inhibitors

Ophiobolin A and E6 Berbamine are specific antagonists of CaM, and thus disrupt the translocation competence of small-secreted proteins (SSPs) that depend on CaM during the targeting phase towards the ER [71].

## Driving forces in post-translational translocation

The polymerization of peptides in the ribosome provides a directional driving force during co-translational translation. GTP is hydrolyzed during the ribosomal chain elongation step, which pushes the preprotein through the ribosomal exit tunnel and translocon pore [73] (Fig. 1). However, roughly 70 residues still remain inside the tunnel and channel after completion of translation [74] and this chain can move freely up and down through the channel. Cells employ a secondary mechanism to complete the translocation of this free-moving substrate: after sufficient downwards diffusion (due to Brownian motion) of the chain through Sec61 and towards the lumen, the chaperone BiP can bind to the exposed chain segment. Once bound to BiP, the chain segment cannot diffuse back to the cytosolic side. This starts a cycle, where stepwise binding of additional BiP molecules acts as a ‘molecular ratchet’ that pulls nascent chains towards the lumen [75, 76] (Fig. 1).

Sec61-dependent post-translational translocation (Fig. 4) requires the Sec62/63 complex and the lumenal BiP chaperone for directional protein movement through the translocon channel towards the lumen [77], similar to the function of BiP in co-translational translocation. Sec62 is an integral membrane protein that forms a stable complex with Sec63 and associates with ER-bound ribosomes, where it binds near the ribosomal exit tunnel [78] (Fig. 1). These proteins are highly abundant in the mammalian ER [79]. A study by Reithinger et al. used the yeast model system to show that Sec62 is required in addition to SRP for the targeting and translocation of uncleaved signal anchor sequences with moderate hydrophobicity [80]. Sec62 is also required for post-translational translocation of SSPs, an important class of SRP-independent proteins [77]. Due to their small size (< 160 aa), the signal peptide of these SSPs remains (partially) buried inside the ribosomal exit tunnel after completion of translation, which prevents recognition by SRP [81]. Additionally, Sec62 is suggested to function as a receptor that detects Ca^2+^ leakage through Sec61 and facilitates CaM recruitment [82], which in turn mediates Ca^2+^-dependent closure of the channel [83]. Interestingly, the different functional studies and the location of Sec62 near the ribosome suggests that both (co- and post-) translocational systems overlap at this site.

### The BiP chaperone has multiple functions during both co- and post-translational translocation

ER-resident Hsp40/DnaJ-like proteins ERdj1 and Sec63 (ERdj2) are integral membrane proteins that recruit the lumenal Hsp70 member BiP (termed Kar2p in yeast) to the translocon (Figs. 1, 4a) [77, 84–86]. BiP consists of a substrate binding domain and a nucleotide-binding domain and can perform several functions during protein translocation: (1) BiP is proposed to drive translocon gating from the closed to the open state during early stages of translocation, as it was found to assist with insertion of precursor peptides into the channel [87]. The chaperone can bind to loop 7 of Sec61α, which forms the hinge region between both halves of the channel, and this binding energy facilitates insertion of ‘weak’ nascent peptides which are otherwise unable to open the channel by themselves [7, 88]. (2) It closes off the lumenal side of Sec61 and returns the channel to a closed state to prevent Ca^2+^ efflux after translocation has ended [88, 89]. (3) BiP acts as a molecular ratchet to complete translocation of pre-proteins in both co- and post-translational Sec61-dependent translocation (Figs. 1, 4a) [76, 90]. The substrate-binding domain of BiP has affinity for unfolded hydrophobic oligopeptides [91] and this affinity is regulated by its nucleotide-binding domain. The ATP-bound state of BiP has low substrate affinity while the ADP-bound state has high affinity [92]. Recruitment of BiP to the translocon complex allows Sec63 and ERdj1 to activate BiP’s ATPase activity with their lumenal J domain. This converts the bound ATP to ADP and thus increases the substrate binding affinity, which allows BiP to bind peptides as they emerge from the translocon. After completion of translocation, release of bound proteins from BiP requires exchange of ADP for ATP, which is performed by two lumenal nucleotide exchange factors: Grp170 and Sil1 [92].

### SecA-dependent post-translational translocation

Bacteria (and chloroplasts in plants) uniquely contain SecA as part of their translocation systems. SecA recognizes targeting signals in cytoplasmic pre-proteins and functions as an essential motor protein that mechanically drives post-translational translocation, as it uses sequential cycles of substrate clamping and ATP-dependent domain movement to push the preprotein through the SecYEG channel [14, 93]. Interestingly, mature protein regions are also bound to a (currently unknown) site on SecA and can target the preprotein independent of their signal peptide, but this only occurs after allosteric activation of SecA by a bound signal peptide [94, 95].

## Inhibition of the translocational driving force

### Mycolactone

In addition to the previously described actions, mycolactone also depletes BiP and this may affect the translocation of other proteins indirectly [49]. Co-translational translocation of BiP itself could be inhibited by mycolactone too, resulting in lower luminal BiP levels, and further amplifying the inhibition of translocation for BiP-dependent substrates.

### Valinomycin

The macrocycle valinomycin, isolated from an Actinomycete culture, is a known potassium anionophore [96]. Interestingly, it was identified as a down-regulator of BiP expression and induces cell death in cancer cells with glucose starvation [97]. The chaperone function of BiP protects cells during ER stress and supports the unfolded protein response. Reduced BiP levels in valinomycin-treated cancer cells can thus result in cell death under ER stress conditions, which are typical for solid tumor environments where nutrient supply is limited due to the poor vascularization. Valinomycin is also a signal peptide-specific inhibitor of hamster prion protein (PrP) translocation [98]. Inefficient translocation of PrP leads to cytosolic accumulation and misfolding of the protein, resulting in cytotoxic protein aggregates. Interestingly, human PrP was not affected by valinomycin and this selectivity was due to small differences in the signal peptides of these two homologous proteins. The downregulation of BiP is suggested as a mechanism for the selectivity of the compound, as BiP dependency is linked to targeting properties of the signal peptide: ‘weak’ signal peptides require assistance from accessory factors for translocon gating, which is one of the proposed functions of BiP.

### R/1 derivatives

Two derivatives of R/1, MAL3-39 and MAL3-101, were shown to inhibit the in vitro post-translational translocation of ppαF substrates, but these derivatives are less active and only inhibit, respectively, 45 and 30% of translocation at 300 µM [99]. While R/1 affects both the innate and Hsp40-stimulated ATPase activity of Hsp70 chaperones, MAL3-39 and MAL3-101 only inhibit the J domain-mediated stimulation of Hsp70 ATPase activity. It is suggested that these two derivatives enter the ER lumen, where they modulate the functions of BiP/Kar2p and Sec63, and therefore, affect post-translational translocation.

### Bactericidal ATPase inhibitors

In bacteria, most secretory and membrane proteins (~ 95% in *Escherichia coli*) rely on the highly conserved Sec pathway for their membrane insertion or translocation across the plasma membrane [100]. SecA is essential for SecY-dependent post-translational translocation in bacteria (Fig. 4b), but it is absent in eukaryotes, which makes this an attractive target for the development of new antibacterial drugs (Table 1).

**Table 1:** Overview of different inhibitors and modulators of translocation

Inhibitor	Target	Affected translocation pathways
Virulence factors and retrograde transport inhibitors		
Eeyarestatin I	Prevents NC transfer from SRP to Sec61 [24] Binds p97 ATPase [23, 126]	Sec61-dependent co-translocational import [24] ER-associated protein degradation [23, 127] Induces UPR [25]
Mycolactone	Induces an irreversible conformational change in Sec61α [31]	Broad effect on Sec61-dependent co-translational translocation, selective inhibition of Sec61-dependent post-translational translocation. SSPs are less affected. [29]
Exotoxin A	Modulates translocon gating, binds IQ motif on Sec61α N terminus, similar to CaM [33, 34]	ER retrotranslocation of immunogenic peptides [32]
Chaperone inhibitors		
NSC 630668-R/1	Hsp70 ATPase inhibitor (BiP/Kar2p) [72]	Post-translational translocation in yeast ER [72]
MAL3-39	J domain-mediated Hsp70 ATPase activity [99]	Post-translational translocation in yeast ER [99]
MAL3-101	J domain-mediated Hsp70 ATPase activity [99]	Post-translational translocation in yeast ER [99] VSG_117 transport into *Trypanosoma brucei* ER [119]
E6 Berbamine	Calmodulin antagonist [128]	Calmodulin-dependent post-translational translocation of small proteins [71]
Ophiobolin A	Calmodulin antagonist [129]	Calmodulin-dependent post-translational translocation of small proteins [71]
SecA inhibitors		
Equisetin and CJ-21058	SecA ATPase inhibitor [101]	SecA-dependent post-translational translocation [101] VSG_117 transport into *Trypanosoma brucei* ER [119]
Rose bengal and erythrosin B	SecA ATPase inhibitor [102]	SecA-dependent post-translational translocation [102]
P97-A4, P87-A4, 17D9, P91-E9, 16F6	Inhibits signal peptide binding to SecA [104]	SecA-dependent post-translational translocation [104]
Bisthiouracil	SecA ATPase inhibitor [105, 106]	SecA-dependent post-translational translocation [105, 106]
SCA-21	SecA ATPase inhibitor [107]	SecA-dependent post-translational translocation [107]
Cyclic depsipeptides and triaza compounds		
HUN-7293	Traps NC TMDs at the cytosolic side of the Sec61α lateral gate [47]	ICAM-1 and VCAM-1 translocation [35]
Cotransin	Traps NC TMDs at the cytosolic side of the Sec61α lateral gate [47]	Signal-peptide-specific inhibition of ER translocation: VCAM-1, P-selectin, angiotensinogen, β-lactamase, CRF-R-1 [36], endothelin B receptor [37], TNFα [39], HER3 [38] Affects most secreted proteins, but only a minority of integral membrane proteins [40]
CAM741	Prevents correct insertion of VCAM-1 NCs into the translocon [62]	VCAM-1 [61] and VEGF [63] translocation
Apratoxin A	Stabilizes the Sec61α in a closed conformation [48]	Selective inhibition of co-translational translocation [45] Broad-spectrum inhibition of translocation [48]
Decatransin	Targets Sec61/SecY, similar but not identical to cotransin [41]	Sec-dependent co- and post-translational translocation [41]
Valinomycin	K^+^ anionophore [96] Down-regulates BiP [97]	Signal-peptide-specific inhibitor of hamster PrP translocation [98]
CADA	Interferes with SP topology inversion inside the Sec61 translocon [54]	Co-translational translocation of human CD4 [54] and sortilin [58]

CJ-21058, a derivative of the fungal antibiotic equisetin was discovered as the first inhibitor of *E. coli* SecA ATPase activity [101]. It also showed antibacterial activity against multidrug-resistant *Staphylococcus aureus* and *Enterococcus faecalis*. The fluorescein analogs rose Bengal and erythrosin B are able to inhibit the in vitro translocation of proOmpA through inhibition of the SecA ATPase activity. These compounds are predicted to occupy the ATP binding site in SecA [102]. Inhibition of ATPase activity was indeed competitive at low ATP concentrations, however, more recent data shows that rose bengal non-competitively inhibits the translocation activity of SecA at high ATP concentrations [103]. They have both bacteriostatic and bactericidal effects. Five compounds (termed P97-A9 family in Fig. 4) were identified in a small molecule screening as inhibitors of SecA translocase activity [104]. These compounds target the signal peptide binding site in SecA, a conserved and essential feature in SecA from both Gram positive and Gram negative bacterial species, and also showed weak antimicrobial activity. Recently discovered analogues of bisthiouracil [105, 106] and the bistriazole compound SCA-21 [107] are more potent inhibitors of the ATPase activity and SecA-dependent protein translocation. Moreover, they are effective against methicillin-resistant *S. aureus* strains. For most of these SecA inhibitors, permeability of the outer membrane in Gram negative bacteria was required for the antimicrobial activity [108]. These novel antibacterial mechanisms are promising solutions for the urgent problem of multidrug-resistant pathogens.

## Alternative targeting pathways

### Tail-anchored proteins

**Fig. 5:**
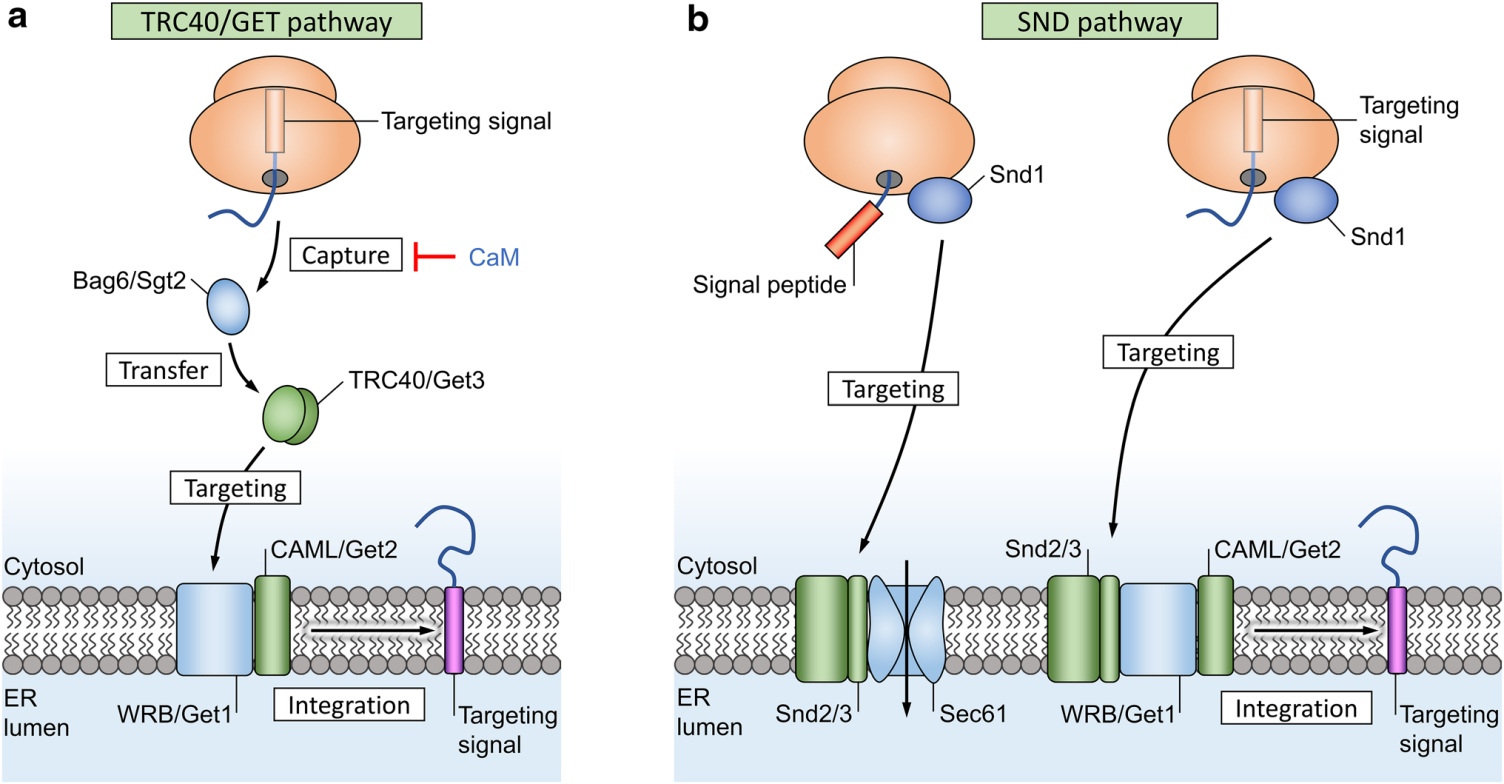
Alternative targeting pathways for translocation. **a** Tail-anchored proteins have a single transmembrane domain at their C terminus that also acts as a targeting signal (purple box). A pretargeting complex captures these C-terminal signals after release from the ribosome and transfers them to TRC40 (or Get3 in yeast) for targeting to the membrane receptor WRB/CAML (Get1/Get2 in yeast). The targeting factor TRC40 operates as a dimer and requires ATP to transition between open and closed states. Inhibitors of translocation are indicated in blue text. **b** The SRP-independent (SND) pathway serves as a backup system for the classical Sec61 and TRC40 targeting pathways. The membrane proteins Snd2 and Snd3 function as targeting receptors for Snd1

Due to their structure, TA proteins should not be able to use the SRP-driven targeting system, as their C-terminal targeting signal remains hidden inside the ribosome during protein synthesis (Fig. 5). Instead, they rely on the Bag6/SGTA complex (or Sgt2 in yeast) and a completely different set of proteins termed transmembrane domain recognition complexes (TRCs) for recognition and targeting to the ER membrane receptors WRB and CAML. These protein complexes then facilitate membrane insertion independent of Sec61 [109]. The yeast ortholog of TRC is GET; “guided entry of tail-anchored proteins”.

### SRP-independent targeting

A route that functions in parallel with the SRP and TA pathways, termed SND (SRP-independent targeting), was recently discovered in *Saccharomyces cerevisiae* [110, 111]. Three proteins were identified (Fig. 5): Snd1 is located in the cytosol and predicted to interact with the ribosome, where it may act as the receptor for hydrophobic targeting signals. Snd2 and Snd3 form a complex in the ER membrane, together with the translocon, and could act as targeting receptors. Though only described in yeast, a human ortholog of Snd2 exists (hSnd2) and its function as a membrane receptor was recently confirmed [112]. The SND pathway was originally shown to serve as a backup targeting system for both SRP-dependent and TRC40-dependent pathways in yeast [110]. Recent evidence also showed that the TRC40 targeting pathway is not essential for integration of TA proteins in human cells, as membrane integration of TA proteins in TRC40 knockouts can be complemented by both the SND and SRP pathways [113]. This supports the idea that SRP is likely able to recognize some targeting signals inside the ribosomal exit tunnel, near the end of TA protein translation, instead of outside the ribosome [114–116].

### Inhibitors of the TRC40 pathway

Calmodulin inhibits ER membrane insertion of mammalian TA proteins in multiple translocation pathways, likely due to binding of CaM to the targeting signals as in Sec-dependent post-translational translocation, masking them for recognition. This function of CaM has been suggested as a regulatory mechanism for the TRC40 pathway [117].

TRC40 is also involved in ER targeting of SSPs [118] and glycosylphosphatidylinositol (GPI)-anchored proteins [66]. Targeting of these proteins is thus apparently facilitated by multiple SRP-independent pathways. Mycolactone is an interesting inhibitor in this regard because it only partially affects translocation of SSPs. Some SSP pre-proteins that normally use the Sec61 pathway are able to escape the translocational block; in the presence of mycolactone, they are redirected to alternative pathways such as the TRC40 pathway. Importantly, hydrophobicity of the signal peptide and properties of the mature domain were shown to affect mycolactone sensitivity of SSPs [31].

### Species-specific signal peptides

The GPI-anchored surface protein VSG_117 protein from *Trypanosoma brucei*, a protozoan parasite responsible for human African trypanosomiasis (sleeping sickness), is used for immune evasion in the bloodstream. Interestingly, post-translational translocation of VSG_117 is inhibited by MAL3-101, equisetin and CJ-21058 [119], three ATPase-related inhibitors (Table 1).

Trypanosomatida diverged early from other eukaryotes and they developed a surprisingly different SRP complex [120]. Importantly, *T. brucei* signal peptides are incompatible with the eukaryotic post-translational translocation pathway [121]. Properties of the hydrophobic region of the targeting signals appear to determine compatibility with the mammalian translocation system, likely due to their different interactions with the unique trypanosomal SRP. Most trypanosomal signal peptides are also able to use multiple translocation pathways, but they depend heavily on the SRP-independent post-translational translocation pathway for the production of their GPI-anchored proteins. Sec71, a non-essential post-translational translocon component in yeast which is not present in mammalian cells, is also present in *T. brucei* and essential for their survival [122].

Compounds like MAL3-101 might, therefore, offer a novel method for the treatment of trypanosomal infection. Selective inhibition of only trypanosomal post-translational translocation is possible due to the differences in the composition of host and parasite signal peptides and the corresponding translocon complexes. This key therapeutic concept of signal peptide-selective translocation inhibition was previously demonstrated in mammalian cells for the HUN-7293/cotransin family and CADA compounds (Table 1).

## Conclusions

Since the discovery of the ubiquitous Sec-dependent protein translocation pathway, various translocation inhibitors have been discovered (Table 1). Furthermore, recent reports have uncovered a significant redundancy between the different protein translocation pathways, in which properties of the targeting signals determine the preference for the selected translocation system. In this review, we highlighted how multiple stages in the different translocation pathways can be modulated or even inhibited, leading to either selective or broad inhibition of protein translocation. In general, most of the translocation inhibitors described here affect the recognition, chaperoning or function of the signal peptides.

Based on the characteristic inhibitory profile of each translocation inhibitor, there is some optimism that modulation of protein translocation can be exploited for the development of new therapeutic and antimicrobial applications. Recent CRISPR/Cas9-based screenings of host factors involved in viral replication revealed an important role of translocon-associated components [123, 124]. For cotransin, it has already been shown that it limits proteostasis of enveloped viruses such as influenza virus, human immunodeficiency virus and dengue virus [125]. In addition for mycolactone, one might expect a similar broad antiviral effect as it influences translocation of a broad range of proteins. Screening for translocation inhibitors has resulted in the discovery of eeyarestatin I and apratoxin A with anticancer properties, and a new class of broad-spectrum antibacterial compounds is being developed based on inhibition of SecA. Notwithstanding the long road to go for translocation inhibitors to become therapeutics, in the mean time they are valuable as research tools to decipher the remaining mysteries of protein translocation across membranes.

## Abbreviations

BiP: Binding immunoglobulin protein
CADA: Cyclotriazadisulfonamide
CaM: Calmodulin
CRF-R-1: Corticotropin-releasing factor receptor 1
ER: Endoplasmic reticulum
ERAD: Endoplasmic reticulum-associated protein degradation
ESI: Eeyarestatin I
GET: Guided entry of tail-anchored proteins
GPI: Glycosylphosphatidylinositol
hCD4: Human CD4
HHV-7: Human herpes virus-7
HIV: Human immunodeficiency virus
Hsc70: Heat shock cognate 70
Hsp70: Heat shock protein 70
ICAM-1: Intercellular adhesion molecule 1
NC: Nascent chain
OST: Oligosaccharyl-transferase complex
PMF: Proton motive force
ppαF: Pre-pro-α-factor
*prl*: Protein localization
PrP: Prion protein
RNC: Ribosome-nascent chain complex
SA: Signal anchor
SND: SRP-independent targeting
SP: Signal peptide
SPC: Signal peptidase complex
SR: SRP receptor
SRP: Signal recognition particle
SSP: Small secreted protein
TA: Tail-anchored membrane protein
TMD: Transmembrane domain
TNFα: Tumor necrosis factor alpha
TRAM: Translocating chain-associated membrane protein
TRAP: Translocon-associated protein
TRC: Transmembrane domain recognition complex
UPR: Unfolded protein response
VCAM: Vascular cell adhesion molecule
